# Vessel wall differences between middle cerebral artery and basilar artery plaque**s** on magnetic resonance imaging

**DOI:** 10.1038/srep38534

**Published:** 2016-12-05

**Authors:** Peng-Peng Niu, Yao Yu, Hong-Wei Zhou, Yang Liu, Yun Luo, Zhen-Ni Guo, Hang Jin, Yi Yang

**Affiliations:** 1 Stroke Center, Department of Neurology, The First Hospital of Jilin University, Xinmin Street 71#, Changchun 130021, China; 2 Department of Radiology, The First Hospital of Jilin University, Xinmin Street 71#, Changchun 130021, China

## Abstract

A recent study showed that posterior circulation plaques have a greater capacity for positive remodeling in a non-Asian population. We aimed to investigate if the features of plaques in the middle cerebral artery (MCA) were different from those in the basilar artery (BA) in a northern Chinese population. We retrospectively analysed the records of 71 consecutive patients with acute ischemic stroke. All patients had at least one MCA or BA plaque with early or mild (<50% stenosis) atherosclerosis identified using vessel wall magnetic resonance imaging. The remodeling ratio, eccentricity index, and plaque range were compared between MCA and BA plaques using multilevel analysis. A total of 101 plaques were included. There were 70 plaques located in the MCA and 31 plaques located in the BA. The features of non-advanced atherosclerotic plaques did not differ between the MCA and BA when accounting for the degree of stenosis or plaque burden in a northern Chinese population. Symptomatic plaques were associated with a higher eccentricity index and smaller plaque range than asymptomatic plaques under the same plaque burden. Further studies are warranted to investigate the progression of atherosclerosis in different intracranial arteries.

Intracranial atherosclerotic disease is one of the important causes of ischemic stroke or transient ischemic attack, especially in Asian, Hispanic and African populations^1^,^2^. Recently, with the rapid development of vessel wall magnetic resonance imaging (VWMRI) techniques, there has been increasing interest in studies of intracranial atherosclerosis. The use of VWMRI has greatly improved the understanding of the pathophysiology of atherosclerosis^3^. The plaque features on VWMRI may be important predictors of stroke and were shown to correlate with pathological findings^4^,^5^,^6^,^7^,^8^.

Atherosclerosis is a systemic disease that may involve several vascular beds, including coronary, peripheral, and renal arteries in addition to the cerebral arteries. However, a previous study showed that the carotid artery has a pattern of remodeling that was distinct from that of the femoral artery, which may be caused by differences in anatomy and blood flow^9^. A recently study also showed that there was a significant difference in arterial remodeling between the arteries of the intracranial posterior circulation and intracranial anterior circulation in a Western population^10^. We aimed to investigate if the features of plaques of the middle cerebral artery (MCA) were different from those of the basilar artery (BA) in the early and intermediate stages of atherosclerosis in a northern Chinese population.

## Results

A total of 71 patients with 101 plaques were included in this study. There were 70 plaques located in the MCA and 31 plaques located in the BA. Among the 70 MCA plaques, 20 were classified as symptomatic, 6 were indeterminate, and 44 were asymptomatic. Among the 31 BA plaques, 11 were symptomatic, 12 were indeterminate, and 8 were asymptomatic (chi-squared test, P for trend = 0.022). Table 1 shows the characteristics of the patients. Table 2 shows the parameters of the MCA and BA plaques.

The inter-observer agreements (intraclass correlation coefficient) for wall area (WA), lumen area (LA), minimum wall thickness, maximum wall thickness, and plaque range were 0.93, 0.92, 0.89, 0.90, and 0.86, respectively.

### Remodeling Ratio

The results of the null model showed that about 38% of the variance in remodeling ratio stems from differences between patients, clearly indicating that a multilevel model was required. The presence of significant between-group heterogeneity (P = 0.035) also indicated that a multilevel model was required (see Supplementary Table S1).

The level 2 explanatory variables of age, sex, hypertension, diabetes mellitus, dyslipidaemia, smoking, and history of coronary heart disease were added to the null model respectively. When these variables were added to the null model, the intercept kept almost unchanged, which suggested that the between-group variation could not be explained by the level 2 explanatory variables.

Level 1 explanatory variables, including the classification of plaque, location of plaque (MCA or BA), and degree of stenosis, were added to the null model. The results showed that the degree of stenosis was significantly associated with the remodeling ratio (P < 0.001) (Table 3 and Supplementary Table S2). The location of plaque (P = 0.768) and classification of plaque were not significantly associated with the remodeling ratio (Table 3 and Supplementary Table S2). The model fit of this model was better than that of the null model (P < 0.001) (see Supplementary Table S3). Testing results of the level 1 random slope coefficients suggested that none of the variables should be set as random slope. Thus, the above model is the final model. About 11% of the variance at level 1 and about 37% of the variance at level 2 can be explained by the final model (Table 3 and Supplementary Table S2).

### Eccentricity Index

The results of the null model indicated that a multilevel model was required for the outcome measures of the eccentricity index (see Supplementary Table S4). The same flow was used to identify the variables that were associated with the eccentricity index. The level 1 explanatory variables of the classification of plaque, location of plaque, and plaque burden were included in the final model. The results showed that the location of plaque (P = 0.387) and plaque burden (P = 0.156) were not significantly associated with the eccentricity index (Table 3 and Supplementary Table S5). Symptomatic plaques were associated with a significantly higher eccentricity index (P = 0.028) than asymptomatic plaques.

### Plaque Range

The results of the null model indicated that a multilevel model was required for the plaque range (see Supplementary Table S6). The level 1 explanatory variables of the classification of plaque, location of plaque, and plaque burden were included in the final model. The results showed that the plaque burden was positively associated with plaque range (P = 0.037) (Table 3 and Supplementary Table S7). Symptomatic plaques were associated with a significantly smaller plaque range (P = 0.042) than asymptomatic plaques.

### Additional Analysis

Eight MCA plaques and two BA plaques were excluded because the degree of stenosis was ≥ 50%. An additional analysis was performed including these 10 plaques. Supplementary Table S8 shows that the results were essentially unchanged.

## Discussion

By using multilevel model, this study found that the remodeling ratio, eccentricity index, and plaque range were not significantly different between MCA and BA plaques with the same degree of stenosis or plaque burden in a northern Chinese population. Symptomatic plaques were associated with a higher eccentricity index and smaller plaque range than asymptomatic plaques.

The multilevel model, also referred to as mixed-effects models, random coefficient models, random effects models, or variance component models, has become a popular method in many fields of research that have nested data structures^11^,^12^. Both statistical and conceptual problems may exist if multilevel model is not considered for data with a multilevel structure. For our study, variables at the plaque level are level 1 variables, which including classification of plaque, location of plaque, degree of stenosis, and plaque range. Variables at the patient level are level 2 variables, which including age and sex. Because more than one plaque from the same patient may exist and be included, and different plaques within the same patient may have similar features, multilevel model should be considered. Furthermore, the results of the null models for the remodeling ratio, eccentricity index, and plaque range in this study showed that a multilevel model is necessary.

By using multilevel model, Qiao *et al*. recently reported that posterior circulation plaques have a higher remodeling ratio than anterior circulation plaques in a non-Asian population^10^, which was inconsistent with our study. However, some important level 1(lesion level) variables such as plaque classification and degree of stenosis were not included in their model. On the other hand, only MCA and BA plaques were included in our study. Furthermore, it is very plausible that some positively remodeled vertebrobasilar arteries could simply be early or developing atherosclerotic “fusiform” aneurysms, which are well known to predominantly affect the posterior circulation. The high remodeling ratio of posterior circulation plaques in the previous study might be attributed to the fact that some of the included posterior circulation plaques may be in the at early or developing stages of atherosclerotic “fusiform” aneurysms.

The formula deduced from the remodeling model (see Supplementary Fig. S1) and data from the plaques (see Supplementary Fig. S2) suggested that the remodeling ratio is positively associated with the plaque burden and inversely associated with the degree of stenosis. Therefore, to investigate if the remodeling ratio differs between MCA and BA plaques, degree of stenosis or plaque burden must be accounted for. Because the degree of stenosis is the most common parameter measured in atherosclerotic lesions, it was included (i.e. adjusted for) in our model. In other words, we determined whether the remodeling ratio was different between MCA and BA plaques with the same degree of stenosis; the results suggest that it was not (Table 3, P = 0.768). A prospective study to investigate the progression of MCA and BA atherosclerosis may further answer the question of whether the remodeling ratio is different between MCA and BA plaques^9^,^13^.

Previous studies showed that positive remodeling is associated with symptomatic plaque; however, this was not confirmed in our study^14^,^15^. Qiao *et al*. reported that culprit plaques were associated with positive remodeling when compared with both non-culprit and indeterminate plaques or non-culprit plaques alone. However, they reported that the association became non-significant after adjusting for the plaque burden^10^. In our study, the remodeling ratio was not associated with the classification of plaque when examined using a one-way analysis of variance (P for trend = 0.909) or multivariate analysis (Table 3). The different methods of plaque classification and/or statistical analysis may account for the controversial result.

Eccentricity is another important feature of atherosclerotic plaques. Previous studies showed that for carotid plaques, larger maximum wall thickness and eccentric plaques were associated with the occurrence of subsequent cerebrovascular events^16^,^17^. For intracranial vascular disease, eccentric wall thickening has been used to distinguish intracranial atherosclerosis from other etiologies^18^,^19^. A recent *in vitro* study showed that eccentric plaques were associated with a non-significant higher risk of cerebrovascular events compared with concentric plaques (30.77% vs. 17.39%; n = 72, P = 0.241)^5^. Our study showed that symptomatic plaques were associated with a significantly higher eccentricity index than asymptomatic plaques after adjusting for the plaque burden (P = 0.028).

We also introduced a new parameter related to plaques, the plaque range. We found that symptomatic plaques had a smaller plaque range than asymptomatic plaques after adjusting for the plaque burden. However, a recent published study showed that plaques with <50% wall involvement were significantly associated with a higher possibility of asymptomatic plaques than plaques with >50% wall involvement^20^. The conflicting results may be attributed to the different methodologies between these two studies. Notably, the plaque burden was adjusted for in our study. The authors explained that plaques with >50% wall involvement are more advanced when compared with plaques with <50% wall involvement^20^, which suggests that the adjustment for the plaque burden is necessary. Another likely explanation for the conflicting results is that the plaque range may be simply acting as a surrogate measure of plaque eccentricity, rather than as a truly independent variable.

There are several limitations of this study. First, a 2D technique may lead to overestimation of thickness measurements because of partial volume effects^10^. Second, because the purpose of this study was to investigate plaque features in the early and mild stages of atherosclerosis, the results of our study may not be generalized to lesions causing ≥50% stenosis. Third, plaque features along the long axis of the artery were not considered in this study. Last and most importantly, the tapering of the vessel was not considered when calculating the parameters of the remodeling ratio, plaque burden, or degree of stenosis. This is a potential source for significant measurement bias, which may have a substantial impact on the results, particularly when the reference slice is distant from the pathological slice.

In conclusion, by using multilevel model, this study found that the remodeling ratio, eccentricity index, and plaque range were not significantly different between non-advanced MCA and BA plaques after controlling for the degree of stenosis or plaque burden in a northern Chinese population. Symptomatic plaques were associated with a higher eccentricity index and smaller plaque range than asymptomatic plaques. A prospective study to investigate the progression of MCA and BA atherosclerosis may further answer the question of whether plaque features are different between MCA and BA plaques.

## Methods

### Study Population

We retrospectively collected consecutive patients who were admitted to the Department of Neurology of the First Hospital of Jilin University between January 2015 and January 2016 with the following characteristics: (1) patients with acute ischemic stroke confirmed by diffusion-weighted imaging within 7 days of symptom onset; (2) patients with atherosclerotic plaques in the MCA or BA that were identified using VWMRI; (3) patients with at least two vascular risk factors^18^; (4) patients without evidence of cardioembolism; and (5) the 2D T2-weighted VWMRI images were of sufficiently good quality to analyse. Only atherosclerotic lesions of the MCA or BA that caused less than 50% stenosis were included. Patients with evidence of moyamoya disease, vasculitis, reversible cerebral vasoconstriction syndrome, or dissection according to the criteria from previous reports were excluded^18^,^19^. The institutional review board of the First Hospital of Jilin University approved this retrospective study. Written informed consent was waived because this was a retrospective study and was approved by the institutional review board. The methods were carried out in accordance with the approved guidelines.

### Imaging Protocol

Since almost all of the VWMRI examinations were performed on a 3 T Siemens Trio MR scanner (Siemens Healthcare, Erlangen, Germany) between January 2015 and January 2016, the only qualifying patient that was examined using the Achieva 3 T scanner (Philips Healthcare, Eindhoven, The Netherlands) was excluded. The VWMRI protocol included 3D time-of-flight MR angiography (repetition time, 21 ms; echo time, 4 ms; slice thickness, 0.50 mm; flip angle, 18°; field of view, 215 mm × 195 mm), 3D T1-weighted SPACE (Sampling Perfection with Application optimized Contrast using different angle Evolutions) (oblique coronal plane acquisition; variable refocusing flip angle; repetition time, 550 ms; echo time, 18 ms; field of view = 210 mm × 210 mm; voxel size = 0.8 mm × 0.8 mm × 0.8 mm; with or without contrast enhancement), and 2D T2-weighted VWMRI (repetition time, 3600 ms; echo time, 56 ms; slice thickness, 2 mm; flip angle, 120°; field of view, 130 mm × 130 mm; 512 × 512 matrix). Two-dimensional T2-weighted VWMRI was obtained perpendicular to the long axis of the BA (approximately 13 slices) or the M1 segments of the relevant MCA (11 slices) and contralateral MCA (11 slices). A 12-channel head coil was used for the above sequences.

### Classification of Plaque

Each plaque was classified as either symptomatic, indeterminate, or asymptomatic according to criteria adapted from previous studies^10^,^14^. A plaque was deemed to be symptomatic if there was an ischemic stroke or transient ischemic attack in the region supplied by the corresponding artery within the preceding four weeks. The plaque should also have been the the only or most stenotic lesion in the corresponding artery. A plaque was deemed to be asymptomatic if there was no history of ischemic stroke or transient ischemic attack in the regions supplied by the corresponding artery. A plaque was deemed to be indeterminate if it was not the most stenotic lesion within the corresponding artery and there was an ischemic events in the regions supplied by the corresponding artery. For patients with a single subcortical infarction in the territory of the MCA, the plaque was classified as indeterminate if the infarction was not in the striatocapsular area^21^. For patients with a single small deep pontine infarction, the plaque was classified as indeterminate^22^.

### Measurement of Plaque

Images from 2D T2-weighted VWMRI were used to perform all measurements. For each atherosclerotic lesion, the cross-sectional slice with the highest degree of stenosis was selected to measure the parameters. For patients with more than one plaque in the same artery, all of the plaques were analyzed. A normal slice (i.e. without plaque on VWMRI) proximal to the selected slice was chosen as the reference site. If there was no such slice, a normal slice distal to the selected slice was chosen as the reference site^14^,^15^,^21^.

**Figure 1:**
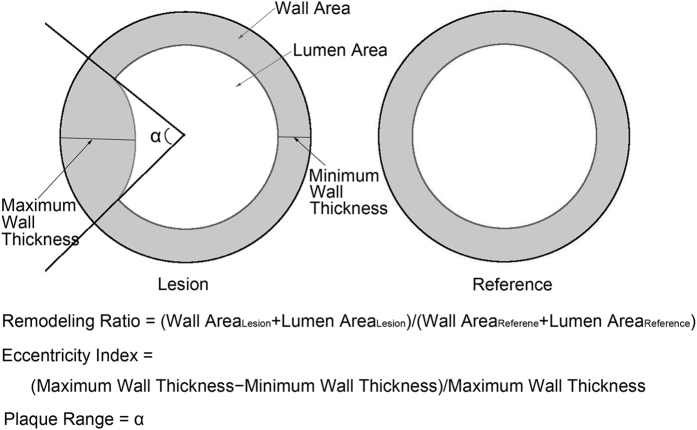
Calculation of the remodeling ratio, eccentricity index, and plaque range.

**Figure 2:**
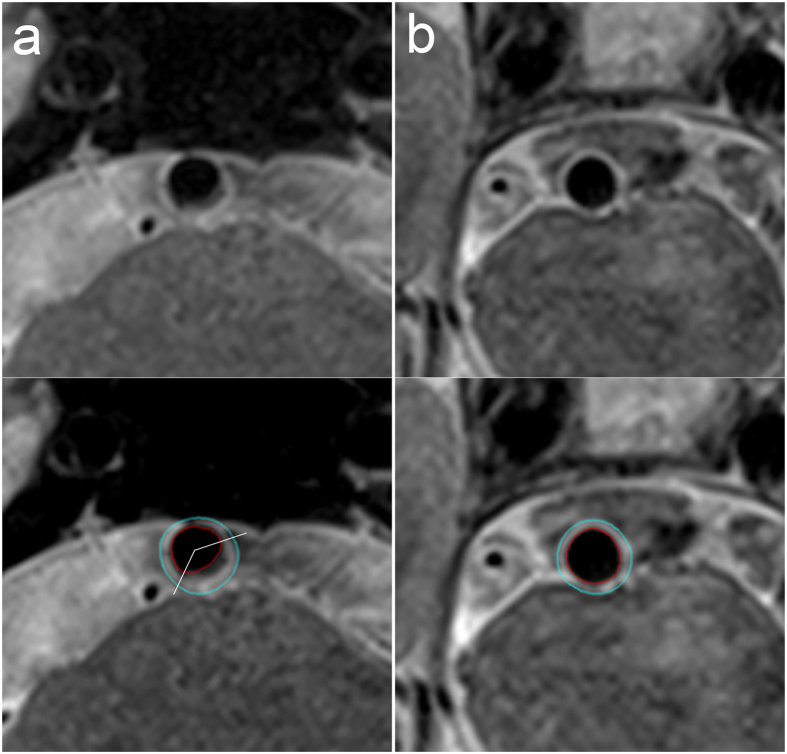
Magnetic resonance images of the vessel wall of a basilar artery plaque. The lumen area and wall area as measured using the MRI-PlaqueView software are 15.03 mm^2^ and 24.21 mm^2^ for the lesion slice (**a**) and 16.04 mm^2^ and 13.85 mm^2^ for the reference slice (**b**). The maximum wall thickness and minimum wall thickness measured using MRI-PlaqueView for the lesion slice are 2.24 mm and 0.75 mm. The distribution range of the plaque measured using RadiAnt DICOM Viewer software is 133.8°. Therefore, the remodeling ratio, eccentricity index, and plaque range for this plaque are 1.31, 0.67, and 133.8°, respectively.

The plaque measurements were performed by two experienced neuroradiologists, each with approximately 3 years of VWMRI reading experience. Quantitative plaque measurement software (MRI-PlaqueView, VPDiagnostics Inc., Seattle, WA) was used to measure the parameters including LA, WA, outer wall area (OWA = LA + WA), minimum wall thickness, and maximum wall thickness (Figs 1 and 2). The lumen counter and outer wall counter of the selected lesion slice and the reference slice were traced in a semi-automated manner by using the software (Fig. 2).

In previous studies, the plaque burden was defined as the ratio of the wall area to the outer wall area^10^. We believed that this ratio or the WA alone would not be appropriate for comparisons of the MCA and BA because the diameter and wall thickness of the normal MCA and BA are different. Consequently, WA_lesion_/WA_reference_ was used as the plaque burden in our study (Fig. 1), a ratio that has been referred to as the wall area index in previous studies^23^,^24^. The arterial remodeling ratio was defined as the ratio of the OWA of the selected lesion site (OWA_lesion_) to the OWA of the reference site (OWA_reference_) (Fig. 1). The eccentricity index was defined as follows: (maximum wall thickness−minimum wall thickness)/maximum wall thickness (Fig. 1)^5^.

We loaded the images of lesions to a DICOM viewer (RadiAnt DICOM Viewer, version 2.2.8; Meixant, Poznan, Poland). The distribution range of the plaque was calculated using the angle tool (Figs 1 and 2). The centre was determined manually based on the normal part of the wall. The edges of the plaque were determined visually.

### Statistical Analysis

Quantitative data are expressed as mean ± SD. Qualitative data are expressed as the number and percentages. For quantitative data, two-sample Student’s t (for normally distributed data) or Mann–Whitney U tests were used. The chi-squared or Fisher’s exact tests were used for qualitative data. The eccentricity index, remodeling ratio, and plaque range were compared between the MCA and BA plaques using multilevel model to adjust for the effect of including more than one lesion from a single patient^10^. The explanatory variables included level 1 explanatory variables (including the classification of plaque, location of plaque, degree of stenosis, and plaque burden) and level 2 explanatory variables (including age, sex, hypertension, diabetes mellitus, dyslipidaemia, smoking, and history of coronary heart disease). Inter-observer agreement for plaque measurements were examined using the intraclass correlation coefficient. The level of statistical significance was set at P < 0.05. SAS 9.3 (SAS institute, Cary, NC, USA) was used to perform the analysis.

## Additional Information

**How to cite this article**: Niu, P.-P. *et al*. Vessel wall differences between middle cerebral artery and basilar artery plaques on magnetic resonance imaging. *Sci. Rep.* **6**, 38534; doi: 10.1038/srep38534 (2016).

**Publisher's note:** Springer Nature remains neutral with regard to jurisdictional claims in published maps and institutional affiliations.

## Supplementary Material

Supplementary Information

## Figures and Tables

**Table 1:** The characteristics of patients.

Patient characteristics (N = 71)	
Age (mean ± SD)	57.45 ± 11.61
Female, N%	16 (22.54%)
Hypertension, N%	57 (80.28%)
Diabetes mellitus, N%	29 (40.85%)
Dyslipidemia, N%	37 (52.11%)
Current smoking, N%	36 (50.70%)
Coronary heart disease, N%	18 (25.35%)
Patients with one plaque, N%	49 (69.01%)
Patients with two plaques, N%	16 (22.54%)
Patients with three plaques, N%	4 (5.63%)
Patients with four plaques, N%	2 (2.82%)
Patients with only MCA plaque, N%	46 (64.79%)
Patients with only BA plaque, N%	21 (29.58%)
Patients with MCA and BA plaques, N%	4 (5.63%)

MCA, middle cerebral artery; BA, basilar artery.

**Table 2:** Parameters of middle cerebral artery and basilar artery plaques.

Parameter*	Middle cerebral artery	Basilar artery^†^	P Value^‡^
Plaque burden	1.25 ± 0.19	1.32 ± 0.19	0.113
Degree of stenosis	0.33 ± 0.14	0.22 ± 0.18	0.007
Remodeling ratio	1.05 ± 0.13	1.11 ± 0.14	0.065
Eccentricity index	0.58 ± 0.07	0.52 ± 0.11	0.010
Plaque range	144.2 ± 34.57	151.9 ± 88.00	0.639

*All values are presented as mean ± SD.

^†^Plaque burden, degree of stenosis, and remodeling ratios are unavailable for three basilar artery plaques because no normal reference slices were available.

^‡^Unadjusted P values.

**Table 3:** Estimates of the final model for the remodeling ratio, eccentricity index, and plaque range.

Parameter	Estimate	Std. Error	T value	P value
Remodeling Ratio				
Intercept	1.1977	0.0412	29.05	<.0001
Plaque location*	−0.0095	0.0317	−0.30	0.7681
Degree of stenosis	−0.3849	0.0811	−4.75	<.0001
Symptomatic plaque	−0.0268	0.0286	−0.94	0.3636
Indeterminate plaque	−0.0031	0.0388	−0.08	0.9368
Asymptomatic plaque^†^	0	—	—	—
Eccentricity Index				
Intercept	0.6113	0.0529	11.55	<.0001
Plaque location*	0.0158	0.0179	0.88	0.3865
Plaque burden	−0.0559	0.0382	−1.46	0.1556
Symptomatic plaque	0.0361	0.0147	2.45	0.0280
Indeterminate plaque	−0.0008	0.0206	−0.04	0.9694
Asymptomatic plaque^†^	0	—	—	—
Plaque Range				
Intercept	87.7430	30.4984	2.88	0.0054
Plaque location*	5.4762	10.2353	0.54	0.5972
Plaque burden	47.3883	21.5642	2.20	0.0371
Symptomatic plaque	−21.2443	9.4811	−2.24	0.0418
Indeterminate plaque	−29.4718	12.3427	−2.39	0.0316
Asymptomatic plaque^†^	0	—	—	—

The restricted maximum likelihood estimation was used.

A significant interaction between the factors was not found.

^*^Middle cerebral artery plaque vs. basilar artery plaque.

^†^Reference for symptomatic plaque and indeterminate plaque.
