# Supplementary material for: Vessel wall differences between middle cerebral artery and basilar artery plaques on magnetic resonance imaging
Source: Sci Rep. 2016 Dec 5;6:38534. doi: 10.1038/srep38534 (PMC5137109; doi:10.1038/srep38534)
Supplement: Supplementary Information [file srep38534-s1.pdf]

Vessel wall differences between middle cerebral artery and basilar artery plaques on magnetic resonance imaging

Peng-Peng Niu, MD[1]; Yao Yu, MD[1]; Hong-Wei Zhou, MD[2]; Yang Liu, MD[2]; Yun Luo, MD[1]; Zhen-Ni Guo, MD[1]; Hang Jin, MD[1]; Yi Yang, MD, PhD[1]*

[1]Stroke Center, Department of Neurology, The First Hospital of Jilin University, Xinmin Street 71#, Changchun 130021, China;

[2]Department of Radiology, The First Hospital of Jilin University, Xinmin Street 71#, Changchun 130021, China.

Supplementary Information:

Supplementary Table S1. Estimates of null model for remodeling ratio.

Supplementary Table S2. Estimates of the final model for remodeling ratio.

Supplementary Table S3. Model fit of final model and null model for remodeling ratio.

Supplementary Table S4. Estimates of null model for eccentricity index.

Supplementary Table S5. Estimates of the final model for eccentricity index.

Supplementary Table S6. Estimates of null model for plaque range.

Supplementary Table S7. Estimates of the final model for plaque range.

Supplementary Table S8. Additional analysis.

Supplementary Fig. S1. Linear regression for remodeling ratio as a function of stenosis and plaque burden. PCC, pearson correlation coefficients.

Supplementary Fig. S2. The relationships among remodeling ratio, degree of stenosis, and plaque burden.

Supplementary Table S1. Estimates of null model for remodeling ratio.

Parameter	Estimate	Std. Error	T/Z value	P value
Fixed Effects				
Intercept	1.0673	0.0156	68.57	<0.001
Covariance Parameters				
Residual	0.0119	0.0033	3.59	<0.001
Intercept	0.0074	0.0041	1.81	0.035

-2LL = -108.7, AIC = -104.7, AICC = -104.6, BIC = -100.2.

ICC = 38.34%.

The restricted maximum likelihood estimation was used.

LL, log likelihood; AIC, Akaike's information criterion; AICC, finite sample corrected version of AIC; BIC, Bayesian information criterion; ICC, intra-class correlation coefficient.

Supplementary Table S2. Estimates of the final model for remodeling ratio.

Parameter	Estimate	Std. Error	T/Z value	P value
Fixed Effects				
Intercept	1.1977	0.0412	29.05	<.0001
Plaque location*	-0.0095	0.0317	-0.30	0.7681
Degree of stenosis	-0.3849	0.0811	-4.75	<.0001
Symptomatic plaque	-0.0268	0.0286	-0.94	0.3636
Indeterminate plaque	-0.0031	0.0388	-0.08	0.9368
Asymptomatic plaque†	0	—	—	—
Covariance Parameters				
Residual	0.0106	0.0032	3.34	0.0004
Intercept	0.0047	0.0036	1.31	0.0956

-2LL = -114.7, AIC = -110.7, AICC = -110.6, BIC = -106.3

Explained variance at level 1: (0.0119 -0.0106)/0.0119 = 0.109. Explained variance at level 2: (0.0074 - 0.0047)/0.0074 = 0.365.

The restricted maximum likelihood estimation was used.

* Middle cerebral artery plaque vs. basilar artery plaque.

†Reference for symptomatic plaque and indeterminate plaque.

LL, log likelihood; AIC, Akaike's information criterion; AICC, finite sample corrected version of AIC; BIC, Bayesian information criterion; ICC, intra-class correlation coefficient.

Supplementary Table S3. Model fit of final model and null model for remodeling ratio.

Parameter	Final Model	Null Model
-2LL	-140.3	-115.2
AIC	-126.3	-109.2
AICC	-125.0	-109.0
BIC	-110.8	-102.4

The lower the better for the parameters.

The -2LL of the final model was small than that of the null model (chi-square value=-115.2-(-140.3) = 25.1, degree of freedom=7-3=4, P<0.001).

The maximum likelihood estimation was used.

LL, log likelihood; AIC, Akaike's information criterion; AICC, finite sample corrected version of AIC; BIC, Bayesian information criterion.

Supplementary Table S4. Estimates of null model for eccentricity index.

Parameter	Estimate	Std. Error	T/Z value	P value
Fixed Effects				
Intercept	0.5540	0.0110	50.59	<0.001
Covariance Parameters				
Residual	0.0027	0.0008	3.52	<0.001
Intercept	0.0064	0.0017	3.76	<0.001

-2LL = -204.7, AIC = -200.7, AICC = -200.6, BIC = -196.2.

ICC= 29.67%.

About 30 per cent of the variance in eccentricity index stems from differences among patients. This clearly indicates that a multilevel model is required.

The restricted maximum likelihood estimation was used.

LL, log likelihood; AIC, Akaike's information criterion; AICC, finite sample corrected version of AIC; BIC, Bayesian information criterion; ICC, intra-class correlation coefficient.

Supplementary Table S5. Estimates of the final model for eccentricity index.

Parameter	Estimate	Std. Error	T/Z value	P value
Fixed Effects				
Intercept	0.6113	0.0529	11.55	<.0001
Plaque location*	0.0158	0.0179	0.88	0.3865
Plaque burden	-0.0559	0.0382	-1.46	0.1556
Symptomatic plaque	0.0361	0.0147	2.45	0.0280
Indeterminate plaque	-0.0008	0.0206	-0.04	0.9694
Asymptomatic plaque†	0	—	—	—
Covariance Parameters				
Residual	0.0018	0.0006	3.01	0.0013
Intercept	0.0049	0.0014	3.38	0.0004

-2LL = -210.2, AIC = -206.2, AICC = -206.0, BIC = -201.7

Explained variance at level 1: (0.0027 -0.0018)/0.0027 = 0.333. Explained variance at level 2: (0.0064 - 0.0049)/0.0064 = 0.234.

The restricted maximum likelihood estimation was used.

* Middle cerebral artery plaque vs. basilar artery plaque.

†Reference for symptomatic plaque and indeterminate plaque.

LL, log likelihood; AIC, Akaike's information criterion; AICC, finite sample corrected version of AIC; BIC, Bayesian information criterion; ICC, intra-class correlation coefficient.

Supplementary Table S6. Estimates of null model for plaque range.

Parameter	Estimate	Std. Error	T/Z value	P value
Fixed Effects				
Intercept	146.37	6.37	22.98	<0.001
Covariance Parameters				
Residual	1927.90	628.36	3.07	0.001
Intercept	1385.88	826.20	1.68	0.047

-2LL = 1092.2, AIC = 1096.2, AICC = 1096.3, BIC = -1100.7.

ICC= 41.82%.

About 42 per cent of the variance in plaque range stems from differences among patients. This clearly indicates that a multilevel model is required.

The restricted maximum likelihood estimation was used.

LL, log likelihood; AIC, Akaike's information criterion; AICC, finite sample corrected version of AIC; BIC, Bayesian information criterion; ICC, intra-class correlation coefficient.

Supplementary Table S7. Estimates of the final model for plaque range.

Parameter	Estimate	Std. Error	T /Z value	P value
Fixed Effects				
Intercept	87.7430	30.4984	2.88	0.0054
Plaque location*	5.4762	10.2353	0.54	0.5972
Plaque burden	47.3883	21.5642	2.20	0.0371
Symptomatic plaque	-21.2443	9.4811	-2.24	0.0418
Indeterminate plaque	-29.4718	12.3427	-2.39	0.0316
Asymptomatic plaque†	0	—	—	—
Covariance Parameters				
Residual	1460.55	343.58	4.25	<0.001
Intercept	139.02	291.71	0.48	0.3168

-2LL = 963.9, AIC = 967.9, AICC = 968.0, BIC = 972.3

Explained variance at level 1: (1927.90 -1460.55)/ 1927.90 = 24.24%. Explained variance at level 1: (1385.88-139.02)/1385.88=89.97%.

* Middle cerebral artery plaque vs. basilar artery plaque.

†Reference for symptomatic plaque and indeterminate plaque.

LL, log likelihood; AIC, Akaike's information criterion; AICC, finite sample corrected version of AIC; BIC, Bayesian information criterion; ICC, intra-class correlation coefficient.

Supplementary Table S8. Additional analysis.

Parameter	Estimate	Std. Error	T value	P value
Remodeling ratio				
Plaque location*	-0.0423	0.0314	-1.35	0.1875
Degree of stenosis	-0.2178	0.0687	-3.17	0.0035
Symptomatic plaque	-0.0116	0.0278	-0.42	0.6819
Indeterminate plaque	-0.0056	0.0397	-0.14	0.8893
Asymptomatic plaque†	0	—	—	—
Eccentricity index				
Plaque location*	0.0252	0.0186	1.35	0.1859
Plaque burden	0.0354	0.0323	1.10	0.2810
Symptomatic plaque	0.0345	0.0154	2.25	0.0372
Indeterminate plaque	-0.0115	0.0219	-0.52	0.6075
Asymptomatic plaque†	0	—	—	—
Plaque range				
Plaque location*	6.2467	10.4012	0.60	0.5526
Plaque burden	61.6742	19.2759	3.20	0.0321
Symptomatic plaque	-23.4035	9.2089	-2.54	0.0205
Indeterminate plaque	-33.2456	12.8160	-2.59	0.0183
Asymptomatic plaque†	0	—	—	—

* Middle cerebral artery plaque vs. basilar artery plaque. † Reference for symptomatic plaque and indeterminate plaque. Ten plaques with ≥ 50% stenosis were included in the final model.

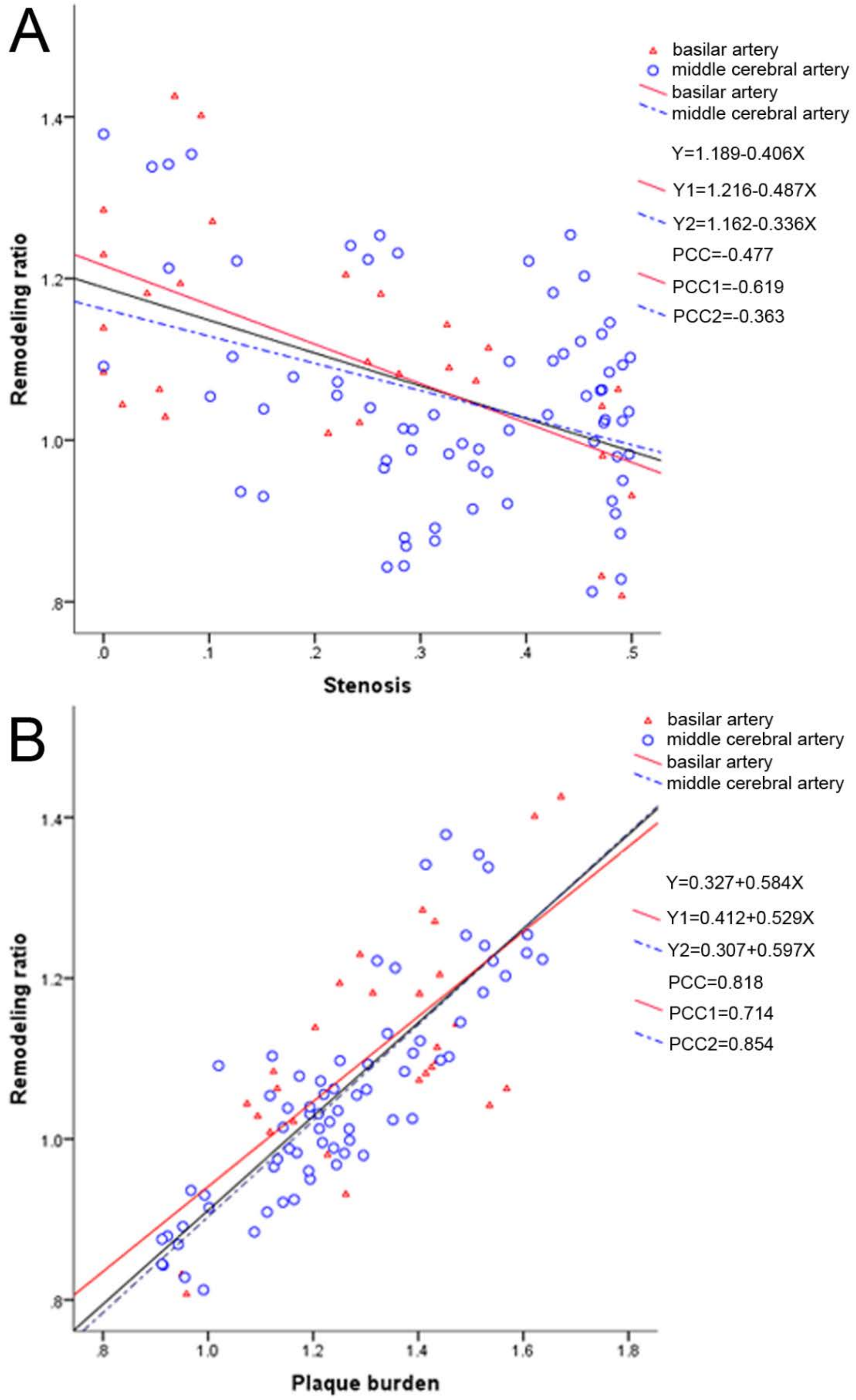

Supplementary Fig. S1. Linear regression for remodeling ratio as a function of stenosis (A) and plaque burden (B). PCC, pearson correlation coefficients.

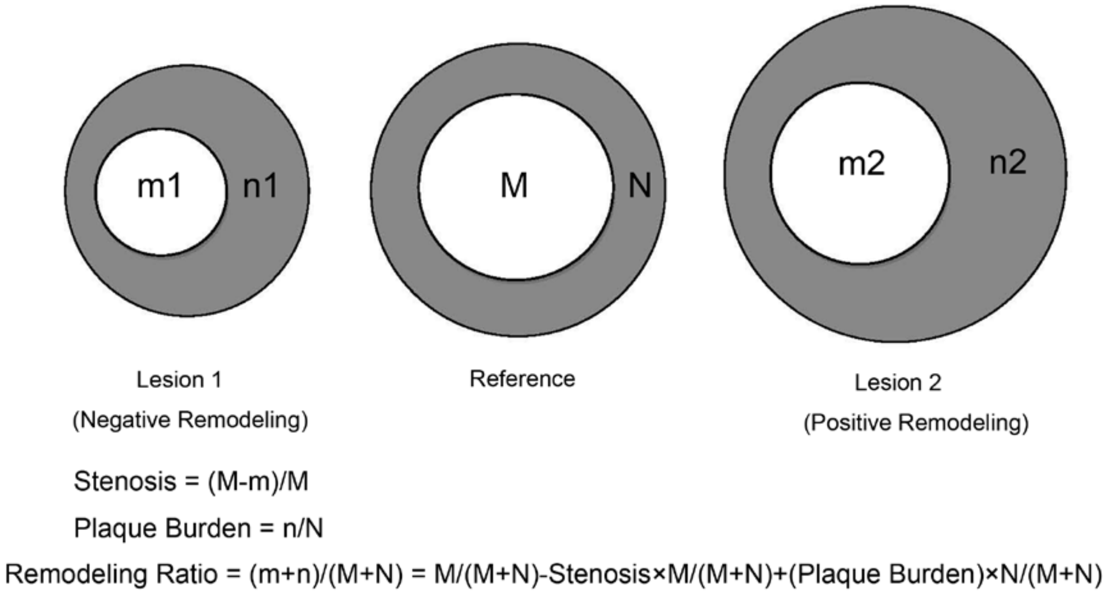

Supplementary Fig. S2. The relationships among remodeling ratio, degree of stenosis, and plaque burden.
